# Reduced beta connectivity during emotional face processing in adolescents with autism

**DOI:** 10.1186/2040-2392-5-51

**Published:** 2014-10-27

**Authors:** Rachel C Leung, Annette X Ye, Simeon M Wong, Margot J Taylor, Sam M Doesburg

**Affiliations:** Department of Diagnostic Imaging, Hospital for Sick Children, 555 University Avenue, Toronto, Ontario M5G 1X8 Canada; Department of Psychology, University of Toronto, 100 St. George Street, 4th Floor, Sidney Smith Hall, Toronto, Ontario M5S 3G3 Canada; Neuroscience & Mental Health, Hospital for Sick Children Research Institute, 555 University Avenue, Toronto, Ontario M5G 1X8 Canada; Institute of Medical Science, University of Toronto, Faculty of Medicine, Medical Sciences Building, 1 King’s College Circle, Toronto, Ontario M5S 1A8 Canada; Department of Medical Imaging, Faculty of Medicine, University of Toronto, 263 McCaul Street - 4th Floor, Toronto, Ontario M5T 1 W7 Canada

**Keywords:** Functional connectivity, Autism spectrum disorders, Affect processing, Neural oscillation, Magnetoencephalography, Social cognition, Neural synchrony, Beta-band, Graph theory, Cognitive development, Face processing

## Abstract

**Background:**

Autism spectrum disorder (ASD) is a neurodevelopmental disorder characterized by impairments in social cognition. The biological basis of deficits in social cognition in ASD, and their difficulty in processing emotional face information in particular, remains unclear. Atypical communication within and between brain regions has been reported in ASD. Interregional phase-locking is a neurophysiological mechanism mediating communication among brain areas and is understood to support cognitive functions. In the present study we investigated interregional magnetoencephalographic phase synchronization during the perception of emotional faces in adolescents with ASD.

**Methods:**

A total of 22 adolescents with ASD (18 males, mean age =14.2 ± 1.15 years, 22 right-handed) with mild to no cognitive delay and 17 healthy controls (14 males, mean age =14.4 ± 0.33 years, 16 right-handed) performed an implicit emotional processing task requiring perception of happy, angry and neutral faces while we recorded neuromagnetic signals. The faces were presented rapidly (80 ms duration) to the left or right of a central fixation cross and participants responded to a scrambled pattern that was presented concurrently on the opposite side of the fixation point. Task-dependent interregional phase-locking was calculated among source-resolved brain regions.

**Results:**

Task-dependent increases in interregional beta synchronization were observed. Beta-band interregional phase-locking in adolescents with ASD was reduced, relative to controls, during the perception of angry faces in a distributed network involving the right fusiform gyrus and insula. No significant group differences were found for happy or neutral faces, or other analyzed frequency ranges. Significant reductions in task-dependent beta connectivity strength, clustering and eigenvector centrality (all *P* <0.001) in the right insula were found in adolescents with ASD, relative to controls.

**Conclusions:**

Reduced beta synchronization may reflect inadequate recruitment of task-relevant networks during emotional face processing in ASD. The right insula, specifically, was a hub of reduced functional connectivity and may play a prominent role in the inability to effectively extract emotional information from faces. These findings suggest that functional disconnection in brain networks mediating emotional processes may contribute to deficits in social cognition in this population.

**Electronic supplementary material:**

The online version of this article (doi:10.1186/2040-2392-5-51) contains supplementary material, which is available to authorized users.

## Background

Autism spectrum disorder (ASD) is a neurodevelopmental disorder in which deficits in social cognition are one of the defining features. The ability to process facial expressions is critically important for social cognition; facial expressions are signals from the social environment and deficits in the ability to accurately perceive and process emotional expressions play a critical role in difficulties with social interactions. While it is generally understood that individuals with ASD experience difficulties with social cues, the current literature on emotional face processing in ASD has yielded inconsistent results, with some authors finding deficits in emotional processing [1–5], whereas others report no deficits [4, 6–8]. Magnetoencephalography (MEG) is a functional neuroimaging technique that allows for the examination of neural network synchronization. We used this approach to investigate the functional network connectivity underlying emotional face processing in ASD, which plays a crucial role in the social cognitive difficulties that are a hallmark of ASD.

Functional magnetic resonance imaging (fMRI) studies have reported atypical activation of social brain networks during emotional face processing in adults with ASD [9–11], while event-related potential and MEG studies have demonstrated abnormal neural responses in youths and adults with ASD, relative to controls [12–16]. Increasing evidence indicates that atypical structural and functional connectivity contribute to cognitive difficulties in ASD. Diffusion tensor imaging (DTI) indicates aberrant white matter connectivity in autism [17] (see [18] for review). Studies of functional interactions among brain areas using hemodynamic neuroimaging (fcMRI) also suggest altered intrinsic and task-dependent network connectivity (see [19] for review). Collectively, such findings lend increasing credence to the view that altered development of brain connectivity may be associated with social and cognitive difficulties in ASD [20–23].

Phase synchronization of neural oscillations among brain areas has been proposed as a mechanism supporting communication in distributed neural networks underlying cognition and perception [24, 25]. Disruption of normal oscillatory network coherence is associated with various neurological and neuropsychiatric conditions [26], and accumulating evidence points to this as being critical for understanding neurodevelopmental disorders, including ASD [27, 28]. Studies of phase coherence among scalp electroencephalography (EEG) electrodes have indicated abnormal functional connectivity in ASD [29]. Reduced interregional MEG synchronization has also been reported in children with ASD during the performance of an executive set-shifting task [30]. Reduced interregional coordination of coupling between low- and high-frequency MEG oscillations has been reported among task-relevant brain areas during a face perception task in ASD [31]. It is not clear, however, whether phase synchronization of neuromagnetic oscillations in distributed networks is also atypical in individuals with ASD during social cognition.

Only a few fMRI studies have examined functional connectivity during affective face processing in ASD. Decreased connectivity between the fusiform gyri, other cortical areas (bilateral posterior cingulate gyri, left cuneus) and subcortical (left amygdala) structures during face processing in adults with ASD, relative to controls, has been observed [32]. Furthermore, atypical connectivity between the right amygdala and frontal and temporal regions has been shown in adolescents with ASD, compared to controls, in response to happy, sad and angry faces [33]. MEG affords both a unique and good combination of spatial and temporal resolution and is a direct measure of neural activity. In addition to both high spatial and temporal resolution, MEG is also silent; an advantage when considering studies in ASD given that sensory issues are known to be a symptom of the disorder. To date, there have been few MEG studies examining functional connectivity during the perception of emotional faces in ASD. One study reported reduced task-dependent local connectivity within the fusiform gyri, which was proportional to reduced long-range connectivity between the fusiform and left precuneus, left inferior frontal gyrus and left anterior cingulate cortex [31]. These results contrast previous hypotheses about increased local functional connectivity at the expense of reduced long-range connectivity [34–36]. Furthermore, despite the importance of studying a paediatric population in order to understand how atypical patterns of connectivity arise in a neurodevelopmental disorder, a limited number of studies has addressed these issues in the developing population and the current literature remains largely focused on adults [32, 33].

Emotional face processing remains an effective method of assessing social cognition in ASD, as the ability to process emotional faces efficiently is integral to social cognition and successful social interactions. Happy and angry facial expressions were chosen as emotional expressions of interest, with the former acting as definitely positive displays of emotion and the latter as negative affective stimuli. Although some studies have reported intact processing of happy emotional information in individuals with ASD [37], investigating the neural connectivity underlying the processing of positive affect in ASD remains an important topic of examination, as individuals with ASD have been shown to have deficits in deriving social reward from happy faces [38]. We also employed angry, rather than fearful, faces as the processing of anger appears to involve a greater understanding of social norms, which has been found to be impaired in many individuals with ASD [39, 40]. Anger, while still considered a basic emotion, is usually exhibited in response to transgressions by another individual and both children with ASD and, to a greater extent, typically developing children can identify social reasons for anger [41]. Furthermore, individuals with ASD have shown difficulty in processing angry faces [42, 43]**.** Hence, focusing on the network connectivity underlying angry face processing in adolescents with ASD is an effective approach for understanding atypical affective processing and, subsequently, social cognitive deficits in ASD.

The present study investigated the perception of both neutral and emotional faces in adolescents with and without ASD to investigate the hypothesis that interregional network synchronization is atypical in ASD during the processing of emotional information in faces. Although the relations between neural connectivity and ASD as measured by oscillatory neural synchronization remain unclear, in light of accumulating evidence indicating impaired long-range connectivity in ASD (see [21] for review), we hypothesized that reduced long-range connectivity in networks related to emotional processing would be observed in adolescents with ASD.

## Methods

### Participants

A total of 22 adolescents with ASD (range: 12 to 15 years, 18 males, mean age =14.2 ± 1.15 years, 22 right-handed) and 17 typically developing controls (range: 12 to 15 years, 14 males, mean age =14.4 ± 0.33 years, 16 right-handed) were recruited. Exclusion criteria for both groups included a history of neurological or neurodevelopment disorders (other than ASD for participants in the clinical group), acquired brain injury, uncorrected vision, colour blindness, IQ ≤65, language skills inadequate for completion of the tasks through self-report and standard contraindications to MEG and MRI. Additional exclusion criteria for controls included use of psychotropic medications; six adolescents with ASD were taking medication at the time of the study. The study was approved by The Hospital for Sick Children Research Ethics Board and written informed consent was obtained from all participants and their parents.

### Qualification measures

A combination of expert clinical judgment, medical diagnostic reports and the Autism Diagnostic Observation Schedule-General (ADOS-G) [44] confirmed clinical diagnoses for all participants with ASD. The mean ADOS-G total score was 10.95 ± 3.37, which was well above the clinical threshold. Full-scale IQ was estimated for all participants using the two-subtest Wechsler Abbreviated Scale of Intelligence, which includes Vocabulary and Matrix Reasoning (WASI-2) [45].

### Magnetoencephalography task

**Figure 1:**
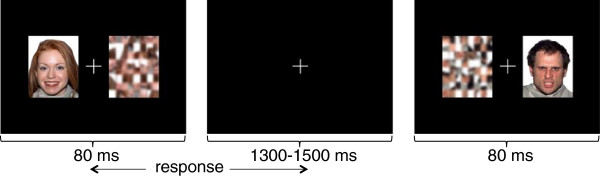
**Implicit emotional face processing task.** An emotional (happy, angry or neutral) face is randomly located in either the left or right hemifield and presented concurrently with a scrambled pattern (target) in the other hemifield with a fixation cross in the center. Participants were instructed to press a button corresponding to the side of the target on a response button box as rapidly as possible.

In each trial of the MEG task, a face (happy, angry or neutral) was presented concurrently with a scrambled pattern on either side of a central fixation cross (Figure 1). Participants were instructed to fixate on the central cross and indicate the location of the scrambled pattern by pressing left or right buttons on a button box as quickly as possible. The MEG task was implicit in that participants were instructed to attend to the scrambled pattern, not the emotional face stimuli. The implicit nature of the task increased ecological validity as adaptive social behaviour requires individuals to automatically and rapidly process affect. A total of 25 colour photographs of different faces (13 males, 12 females) for each of the three expressions were selected from the NimStim Set of Facial Expressions; only happy and angry faces with validity ratings at a minimum of 80% accuracy were selected [46]. To create unique scrambled patterns corresponding to each face, each of the selected faces from the NimStim set was divided into 64 cells, randomized, mosaicked (15 cells per square) and Gaussian blurred (10.0 degrees) using Adobe® Photoshop software. Face-pattern pairs were matched for luminosity and colour.

A total of 50 trials of each of the three expressions in the left and right hemifields (each face was presented twice in each hemifield) were shown in randomized order for a total of 300 trials. Presentation® software (Neurobehavioral Systems) was used to present stimuli. To minimize saccadic eye movements, stimuli were presented for 80 ms with a varying inter-stimulus interval of 1,300 to 1,500 ms; each trial thus ranged from 1380 to 1580 ms. Images were back-projected through a set of mirrors onto a screen positioned at a viewing distance of 79 cm. The visual angle of the stimuli was 6.9° and fell within the parafoveal region of view. Response latency was recorded for each trial.

### Neuroimaging data acquisition

MEG data were recorded using a 151-channel CTF MEG system (MISL, Coquitlam, British Columbia, Canada) at a 600 Hz sampling rate with continuous head localization in a magnetically shielded room at the Hospital for Sick Children. A third-order spatial gradient was used to improve signal quality with a recording bandpass of 0 to 150 Hz. Participants were supine while they completed the experimental paradigm in the MEG. Fiducial coils were placed on the left and right pre-auricular points and the nasion to monitor head position and movement within the dewar. Following the MEG recording, fiducials were replaced by radio-opaque markers for MRI co-registration. A T1-weighted MRI scan (3D SAG MPRAGE: PAT, GRAPPA =2, TR/TE/FA =2300 ms/2.96 ms/90**°**, FOV = 28.8 × 19.2 cm, 256 × 256 matrix, 192 slices, slice thickness =1.0 mm isotropic voxels) was obtained for each participant on a 3 T MRI scanner (MAGNETOM Tim Trio, Siemens AG, Erlangen, Germany) with a 12-channel head coil.

### Atlas-guided source reconstruction

Data epochs (-400 to 400 ms) were extracted surrounding the presentation of happy, neutral and angry faces. MEG data were co-registered with each individual’s MRI image using the three fiducial markers. A multi-sphere head model was constructed for each participant using each individual’s MRI and used to model the forward solution. Statistical Parametric Mapping 2 (SPM2) was used to normalize each individual’s brain space onto a standard Montreal Neurological Institute (MNI) brain. A total of 90 seed locations were then selected, which represent all cortical and subcortical areas in the Automated Anatomical Labeling (AAL) atlas [47]. The coordinates of each seed location were then unwarped from standard MNI space into each individual’s head space. For each subject, broadband time series were reconstructed for each source location and trial using scalar beamformer analysis [48, 49]. Beamformers are based on the concept of adaptive spatial filtering, where the aim is to estimate the signal from a given brain location through the weighted sum of surface field measurements while suppressing signals from all other locations [48, 49]. A weighted sum representing an estimate of activity from the source is created by applying a weight vector to the measurement vector [50]. The spatial filter outputs the activity at the desired source. Contributions from non-target sources are minimized while the power at the desired source is optimized through the least-mean-squares technique.

### Interregional phase synchronization

Data from each epoch were filtered into theta (4 to 7 Hz), alpha (8 to 14 Hz) and beta (15 to 30 Hz) frequency ranges. Alpha, beta and theta band network synchronization was investigated, as these rhythms are understood to be particularly relevant for interregional communication [51–54]. The Hilbert transform was employed to obtain time series of instantaneous phase measures for each trial and source. For each region pair and time point (ms), synchrony was indexed by calculating the phase lag index (PLI) across trials, for each frequency band and subject. This measures the reliability of phase relations between two regions at a given time point, relative to stimulus onset. In this manner, time series representing stimulus locked-phase synchrony were obtained for each region pair, frequency and subject. It should be noted that PLI summarizes the reliability of phase differences across trials for each data point, source pair and frequency, while removing and attenuating synchronization which occurs at or near zero phase difference, and thereby reduces the impact of spurious synchronization originating from common sources [55]. This method produces a source-by-source adjacency matrix for each time point within each analyzed frequency band. These were then averaged across all participants within each group (ASD and control), for each trial condition (angry, happy and neutral). Time series of adjacency matrices were compared across groups. These adjacency matrices, together with average network connectivity time series, obtained by averaging PLI across sources for each time point, were used to investigate task-dependent connectivity dynamics and identify windows for further statistical analyses.

Distinct peaks in task-dependent network connectivity changes were observed in both the time series of adjacency matrices and in the time series of average network connectivity. Specifically, increased beta synchrony was observed in the initial 400 ms following stimulus presentation for all trial conditions (happy, angry and neutral) in both the ASD and typically developing groups. To characterize task-dependent network connectivity dynamics, adjacency matrices representing mean connectivity within this 0 to 400 ms active window were obtained for each subject. Adjacency matrices were then averaged across an equivalent number of time points (-400 to 0 ms) in the pre-stimulus baseline interval.

### Statistical analysis of network dynamics

A data-driven approach was used for the analyses in this study, which was corrected for multiple comparisons, thus *a priori* predictions did not direct analyses. The statistical significance of connectivity differences between the active and baseline intervals, as well as group differences in task-dependent network synchronization, was assessed using Network Based Statistic (NBS) [56, 57]. NBS detects clusters of functionally integrated nodes that significantly differ between groups and is designed specifically for analyses of differences in large-scale networks. NBS accomplishes this by first applying a univariate statistical threshold to each element in the compared adjacency matrix. In this case, to assess task-dependent network connectivity for each group, a t-test is performed comparing the average connectivity during the active window to the average connectivity during the baseline window, for each interregional connection in the 90 × 90 adjacency matrix. The initial univariate threshold for between-group comparisons was adapted for the data distributions being analyzed to T = 4.5, as described by Zalesky *et al*. [56, 57]. This threshold corresponds to a *P* value of *P* <0.0001, while effective control for multiple comparisons is achieved irrespective of this initial threshold [56, 57]. Data surrogation was repeated 5,000 times to create a null distribution, and the size of observed ‘real’ connectivity components was considered relative to the surrogate data distribution in establishing statistical confidence.

Group differences between task-dependent network synchronization were similarly assessed by using time windows identified in the above analysis, subtracting the mean baseline adjacency matrix from the active window adjacency matrix for each subject, and using NBS to assess the statistical significance of group differences. As above, the T statistic for the initial univariate t-test between-groups comparison of individual interregional connections was adapted for the data distributions being compared, as recommended by Zalesky *et al*. [56, 57], to T = 3.5 (equivalent to *P* = 0.0012). The surrogate statistical method described above was used to determine the statistical significance of connectivity components reflecting group differences in task-dependent network synchronization.

### Graph theoretical analysis of dynamic network topologies

Recent application of graph theoretical analysis to structural and functional brain networks has enabled the quantification of the connectivity of given brain regions within larger networks by characterizing connections (edges) between brain areas (nodes) [58]. Particular nodes of interest were identified in a data-driven manner after being identified as playing a critical role in group differences using NBS and graph theoretical analysis. Then, time series of the graph theoretical properties of strength, clustering and eigenvector centrality of the nodes of interest, which were identified in a data-driven manner after being identified as playing a critical role in group differences using NBS and graph theoretical analysis, at each time point were calculated using the Brain Connectivity Toolbox [59] from the adjacency matrices within a given frequency range. Strength indexes how connected a particular node is to all other nodes in the analyzed network, whereas clustering reflects the weight of connections among a node’s neighbours and reflects the degree of functional embeddedness of that node within the network [60, 61]. Eigenvector centrality represents the degree to which a node is a communication hub and also pertains to the importance of that node [62]. Graph theoretical measures were derived from a weighted, non-binarized, undirected connectome as has been used in similar previous studies [30] (see [59] for detailed methods used to derive the connectome). Results obtained using NBS and graph analysis were plotted using the BrainNet Viewer toolbox [63]. This approach of atlas-guided beamformer reconstruction of task-dependent activity, quantification of network synchronization dynamics using PLI, network characterization using the NBS and graph theoretical measures to contrast active and baseline windows, reflects methods that have been previously established [30].

### Behavioural analyses

IQ and response latencies between groups and across emotions on the MEG task were analyzed using SPSS 20.0 software (SPSS Inc., Chicago, Illinois, United States).

## Results

### Beta-band synchronization during face processing

**Figure 2:**
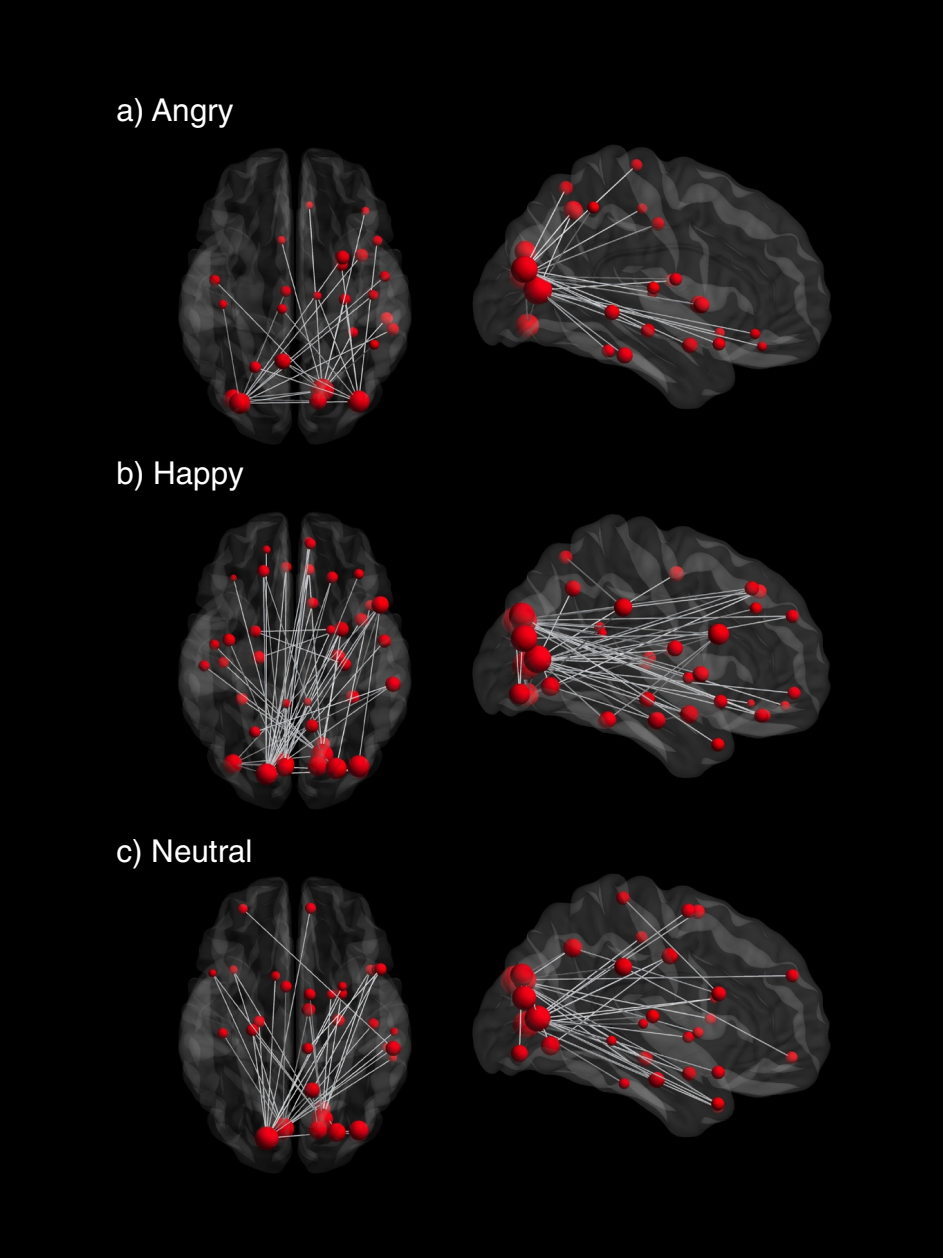
**Task-dependent increases in connectivity across emotions in controls.** Sagittal and axial views of significant task-dependent increases in connectivity identified in **a)** angry, **b)** happy and **c)** neutral conditions in controls. In all three emotions, highly connected brain regions were located in the occipital regions, with a widely distributed network encompassing the frontal, temporal and parietal regions. The size of each region represents its connectivity strength.

Inspection of adjacency matrix time series and mean network connectivity showed increased beta-band coherence in both ASD and control subjects occurring in the first 400 ms following stimulus presentation in all trial conditions. In the typically developing controls, significant task-dependent increases in connectivity were identified in each of the face conditions (Figure 2). Increased beta-band network synchronization involved 26 nodes and 27 edges during processing of angry faces (*P* <0.001, corrected), 41 nodes and 54 edges during processing of happy faces (*P* <0.001, corrected) and 33 nodes and 37 edges during processing of emotionally neutral face stimuli (*P* <0.001, corrected). Across all three emotions, regions expressing strong task-dependent increases in beta-band connectivity strength were located in the occipital areas. Increased beta synchrony was observed in widely distributed networks encompassing the frontal, temporal and parietal regions. Of particular interest were the right fusiform (region 56) and right insula (region 30), which displayed increased beta-band synchronization with other task-relevant brain regions during processing of angry faces (Additional file 1: Figure S1B), whereas the bilateral fusiform and right insula expressed increased beta synchronization with other areas during perception of happy faces. In contrast, in response to neutral faces, neither the fusiform or insula showed increased synchrony with other areas.

### Reduced beta-band synchronization during processing of angry faces in adolescents with ASD

**Figure 3:**
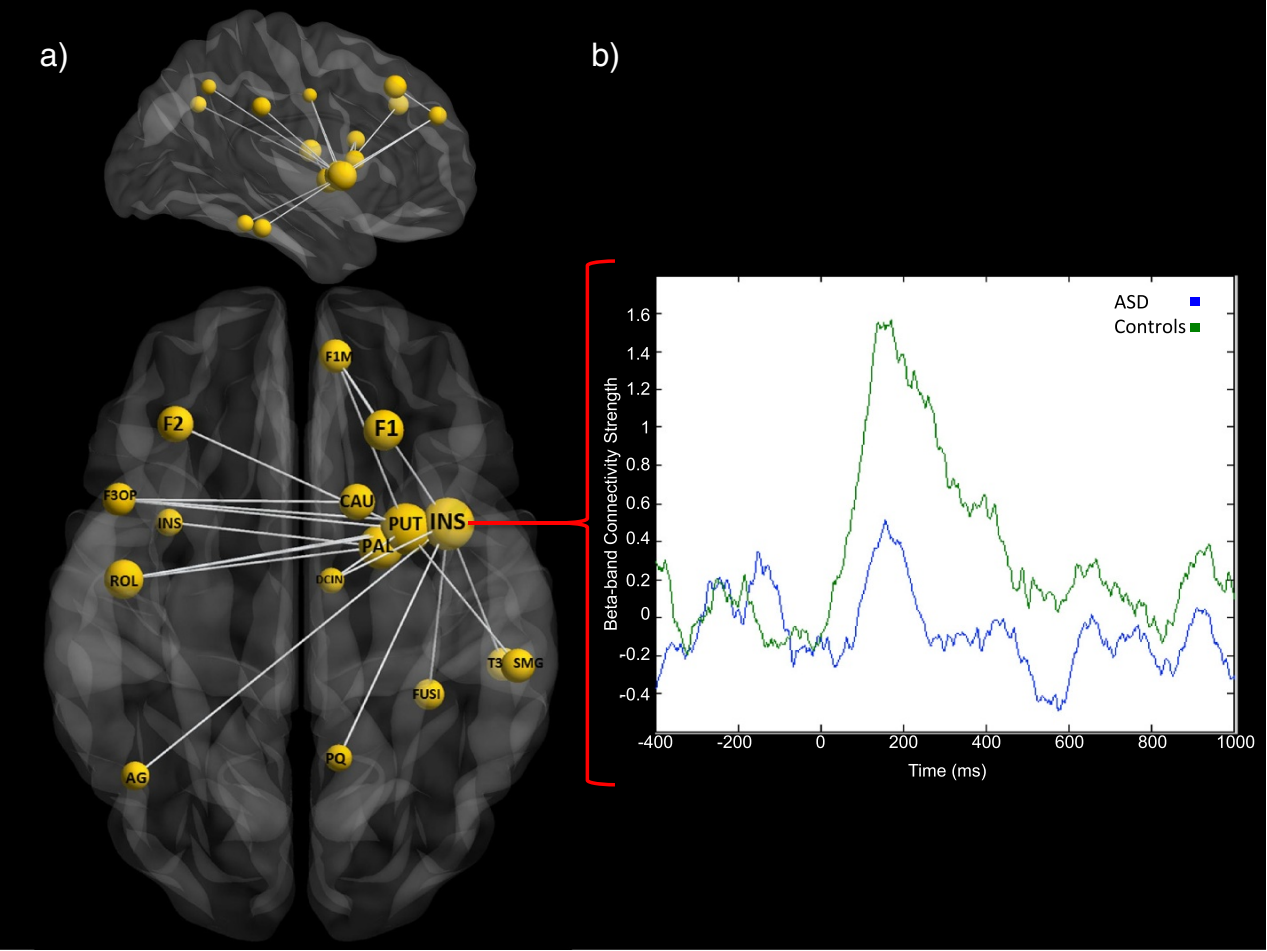
**Between-groups comparison of beta-band interregional phase synchronization during processing of angry faces. a)** Sagittal and axial view of reduced beta-band interregional synchronization in adolescents with ASD in a distributed network during the perception of angry faces. The nodes and edges shown are those identified as expressing reduced beta-band connectivity using NBS. This size of each identified node reflects the magnitude of group differences in connectivity strength. **b)** Between-group comparisons in the right insula showed significantly reduced task-dependent beta-band connectivity strength in adolescents with ASD. This time series was calculated from the strength value for the right insula, from the adjacency matrix for each time point, and subsequently averaged across subjects within each group. See Table 1 for region abbreviations, full region names and MNI coordinates; Left = left, right = right. The size of each region corresponds to its connectivity strength. Inset time series represents the dynamics of right insula connectivity strength during the perception of angry faces for controls (green line) and ASD (blue line), smoothed for clarity. ASD = autism spectrum disorder; MNI = Montreal Neurological Institute; NBS = network-based statistic.

To evaluate group differences in task-dependent connectivity, NBS was employed, revealing reduced beta-band interregional phase-locking in adolescents with ASD (*P* <0.05, corrected), relative to typically developing controls, in a distributed network during the perception of angry faces (Figure 3a, Table 1). This network of reduced connectivity included the right fusiform gyrus and also reduced beta-band synchronization between the right insula and other task-relevant brain regions. No significant group differences were found for the happy or neutral faces, or for other analyzed frequency ranges (*p*s >0.05) Connectivity dynamics during angry face processing in adolescents with ASD and typically developing adolescents were also contrasted across a range of neural regions (Additional file 1: Figure S1, Additional file 2: Table S1).

**Table 1:** **Details of regions showing reduced beta-band interregional phase-locking in ASD during angry face processing**

Region abbreviation	Full region name	MNI coordinates (x, y, z)		
AG	L Angular Gyrus	-44	-61	36
CAU	R Caudate	15	12	9
F2	L Middle frontal gyrus	-33	33	35
F1	R Superior frontal gyrus, dorsolateral	22	31	44
F1M	R Superior frontal gyrus, medial	9	51	30
F3OP	L Inferior frontal gyrus, opercular part	-48	13	19
FUSI	R Fusiform	34	-39	-20
INS	R Insula	39	6	2
	L Insula	-35	7	3
PAL	R Pallidum	21	0	0
PQ	R Precuneus	10	-56	44
ROL	L Rolandic operculum	-47	-8	14
SMG	R Supramarginal gyrus	58	-32	34
T3	R Inferior temporal gyrus	54	-31	-22

### Atypical beta-band insula network topologies in adolescents with ASD

Due to the known importance of the insula for the processing of emotional faces (see [64] for review), we investigated graph properties of beta synchronization involving the right insula. These analyses revealed clear modulation of graph properties for the right insula, as well as differences between adolescents with and without ASD (see Figure 3b). The connectivity strength of this network (Figure 3b), which peaked between 170 and 200 ms in controls, was significantly reduced in ASD. We tested the significance of group differences of insula beta-band graph properties and found significantly reduced task-dependent beta connectivity strength, clustering and eigenvector centrality (all *P* <0.001) in adolescents with ASD. Thus, within this face processing network in adolescents with ASD, the right insula showed significantly reduced task-dependent connectivity to other nodes, reduced functional embeddedness within the network, as well as playing a reduced role as a communication hub, relative to typically developing adolescents (Figure 3b), and this reduction occurred within the first 200 ms following face presentation.

### Behavioural results

Adolescents with ASD (M = 94.69, SD = 14.57) had significantly lower two-sub-test IQ scores than controls (M = 112.47, SD = 11.05), (t(37) = 4.18, *P* <0.001). A three (expression: happy, angry or neutral) × two (group: ASD and controls) ANOVA showed no main or interaction effect between emotion and group on response latency, F(1, 36) = 1.94, *P* = 0.17.

## Discussion

We present the first demonstration of increased neuromagnetic interregional beta-band synchronization during face processing. We also provide the first evidence for reduced interregional beta phase synchronization during emotional face perception in adolescents with ASD, specifically to angry faces. These results are consistent and support previous findings. A reduction in beta phase synchronization only in response to angry faces may reflect the self-conscious nature of anger as an emotion, requiring the understanding of social norms and context, with which individuals with ASD struggle [41, 42, 65–67]. In contrast, seemingly typical processing of happy affect in individuals with ASD was observed, which may be attributed to the greater frequency of encountering and familiarity with happy faces [10, 37]. The pattern of normative beta-band synchronization was predominantly characterized by connectivity between visual brain areas and ‘higher-order’ processing areas. This likely represents the transfer of information from brain areas relevant for vision to other task-relevant regions, including those responsible for face perception and affective processing. The pattern of reduced connectivity in adolescents with ASD did not reflect the normative pattern of task synchronization, but rather indicated reduced synchrony involving regions relevant for face perception and affective processing (the fusiform and insula, respectively). Accordingly, we interpret reduced task-dependent network synchronization in ASD as reflecting reduced communication in brain networks that contribute to social cognition. The fact that this reduction occurred within the first 200 ms and involved the fusiform and insula, as well as association areas such as the supramarginal gyrus and frontal areas, demonstrates the very rapid and complex processing of emotional faces is impaired in adolescents with ASD.

While the ability to perceive basic emotions is largely in place by six years of age, accuracy in discriminating between facial expressions, as well as recognition of more complex emotions, develops with age, continuing into the adolescent period [68–72]. Maturation of emotional processing for unique emotions is staggered across development, with the ability to identify happy expressions maturing earliest while accurate identification of negative emotions, such as anger and fear, mature at later ages, suggesting that negative emotions require more complex processing [70]. Evidence that different emotions develop distinctly is found in reports of a significant increase in sensitivity towards anger from adolescence to adulthood, in contrast to fear, which increases linearly from late childhood into adulthood [73]. A developmental phase at around 11 years of age has been found, during which emotional processing abilities undergo marked improvement, suggesting greater demands on neural areas implicated in emotional processing in early adolescence [74]. The present results suggest that the adolescents with ASD are still impaired in the acquisition of the more difficult emotional expression, anger.

The reduced task-dependent synchronization to angry faces included the right fusiform gyrus, and in particular synchronization between the right insula and other brain areas. Further investigation of task-dependent beta connectivity involving the right insula using graph analysis revealed reduced task-dependent connectivity strength, clustering and eigenvector centrality in adolescents with ASD. These results indicate that ASD may be associated with a reduced ability to marshal the very rapid and complex network communication of brain regions critical for emotional processing; such functional disconnection may contribute to deficits in emotional face processing prevalent in this population. Cognitive and perceptual performance requires selection and functional integration of task-relevant neuronal populations, which may be distributed across brain areas [75]. Multiple lines of evidence indicate that neural synchronization is a mechanism mediating such communication dynamics in the brain [25, 28]. The dynamics of neural synchronization supporting various cognitive and perceptual processes can be effectively imaged in source space using MEG [76]. It is also clear that such task-dependent neuromagnetic synchronization is relevant for understanding individual differences in cognitive ability [77], as well as cognitive difficulties in clinical child populations [78].

Previous MEG research has implicated beta-band connectivity in visual perception [79]. Recent theories synthesizing results across studies and modalities have suggested that beta-band connectivity is particularly pertinent for establishing long-range communication among brain regions, and especially relevant for feedback interactions among brain areas [51, 80]. In light of this, the reduced beta synchronization in the ASD group in the present study could reflect inadequate re-entrant processing in task-dependent networks, involving areas such as the right insula, leading to an inability to effectively extract emotional information from faces.

Current literature implicates the insula as an interface between the frontal and limbic regions, playing a role in assigning emotional salience to perceived events (see [64] for a review) [81–83]. Furthermore, atypical insula activity in individuals with ASD may underlie difficulties in emotional awareness of one’s own self, as well as others [84]. The anterior insula is recruited as part of a larger valence network responsible for attributing emotional salience [85], and is more likely to be hypoactive during social cognitive tasks in ASD [86]. This region also exhibits functional connectivity with the anterior cingulate cortex, a region widely implicated in emotion processing that also shows hypoactivity during social tasks [86–88]. Decreased intrinsic functional connectivity between both the anterior and posterior insular cortices and other brain regions implicated in emotional processing has been noted in youths with ASD [83]. These findings are in concert with our current results and support the notion that altered beta-band synchronization between the insula and other neural regions is associated with affective processing deficits in ASD.

Of further interest was the insular lateralization in our findings. In typically developing individuals, emotional stimuli have been shown to elicit bilateral anterior and mid-insula activation, with left-hemisphere dominance in the anterior and mid-insula towards positively valenced stimuli, and bilateral activation in both regions towards negative stimuli [64]. Previously, a high degree of coupled activity between the left and right insula regions was reported in fMRI of typical adults [89], while decreased interhemispheric connectivity between these regions has been noted in ASD [90]. Only the right insula was found to be a hub of significantly reduced connectivity in the ASD group, showing reduced connections with areas including the right fusiform, right inferior temporal gyrus and superior frontal regions. Reduced early insula beta-band connectivity with the fusiform is of particular interest given the fusiform’s relevance for face processing; these results are in line with previous reports showing fusiform hypoactivation during emotional face processing in ASD [91].

A popular hypothesis is that reduced long-range functional connectivity and increased local connectivity plays a critical role in ASD [34–36]. A body of literature supports the idea of long-range underconnectivity in ASD, however, a review of fcMRI studies in this field found that studies supporting the underconnectivity theory share methodological similarities that may account for the results [19]. Despite such findings, a recent MEG study examining task-related local connectivity through phase-amplitude coupling in the fusiform regions has found both local and long-range underconnectivity in ASD [31]. Furthermore, this study indicated that the strength of local reduction in connectivity was proportional to the reduction in long-range connectivity and was associated with the severity of autistic symptomology, lending credence to the theory of general underconnectivity in ASD [31]. The present study provides a concordant set of findings, and suggests that reductions in task-dependent long-range connectivity may contribute to difficulties in social cognition in ASD.

A limitation of the present study is that although group differences in beta-band synchronization were observed during the perception of angry faces, our approach of using NBS for characterizing network connectivity differences did not support direct analysis of interactions. Specifically, a group-by-emotion interaction could not be addressed in the current study design. Accordingly, it is possible that adolescents with ASD may express atypical network connectivity during the processing of happy and neutral faces, but that the current investigation was insufficiently powered to reveal these differences. A further potential limitation of the present study was the lack of behavioural corroboration for the observed between-group differences in neural networks. However, comparable performance indicates that differences in brain connectivity cannot be attributed to performance. Studies in adults have also found differences in functional connectivity between those with and without ASD without any behavioural group differences in happy and angry faces [33]. These results suggest that while behavioural performance is comparable, individuals with ASD may be processing emotional faces through the recruitment of alternate neural patterns of functional connectivity. Employing tasks with greater emotional and/or cognitive load may reveal behavioural differences with corresponding neural effects. While the accuracy limits of MEG in localizing deep brain sources are still evolving, multiple studies have shown MEG to be effective at localizing deep sources [88, 92] and interregional connectivity involving deep sources [93], thus our findings in this regard were not unexpected. Lastly, our clinical and control groups were not matched for IQ, with our ASD participants showing significantly lower IQ scores relative to controls. Future studies may examine emotional face processing focusing on individuals with ASD with normal IQs or including IQ-matched controls (see [94] for a discussion of this issue).

## Conclusions

The present study provides the first evidence for reduced MEG beta-band synchronization to emotional faces in adolescents with ASD. During processing of angry faces, we demonstrated reduced task-dependent connectivity in graph theory through measurements of strength, clustering and eigenvector centrality, peaking between 170 and 200 ms in the right insula in the ASD group. These findings suggest that difficulties in emotional face perception in those with ASD may be associated with reduced communication among task-dependent brain regions, particularly involving the right insula; a region critical for affective processing. Future studies should explore these findings throughout childhood and thus study the development of these connectivity patterns associated with emotional face processing in ASD.

## Electronic supplementary material

Additional file 1: Figure S1: Connectivity matrices in the beta frequency band during angry face processing. Group averages of connectivity dynamics in the beta frequency band in response to angry faces during angry face processing in A) adolescents with ASD and B) controls. C) Between-group differences in connectivity dynamics highlights the disorganization in connectivity dynamics in ASD. See Additional file 2: Table S1 for regions, coordinates, and corresponding labels. (PDF 298 KB)

Additional file 2: Table S1: Showing regions, locations, and corresponding labels for connectivity matrices in Additional file 1: Figure S1. (DOCX 24 KB)

## Abbreviations

AAL: Automated Anatomical Labeling
ADOS-G: Autism diagnostic observation schedule-general
ASD: Autism spectrum disorders
EEG: electroencephalography
fMRI: functional magnetic imaging
fcMRI: Functional connectivity magnetic resonance imaging
MEG: Magnetoencephalography
MNI: Montreal Neurological Institute
MRI: Magnetic resonance imaging
NBS: Network-based statistic
SPM2: Statistical Parametric Mapping 2.
