# Supplementary figures and images for: Reduced beta connectivity during emotional face processing in adolescents with autism
Source: Mol Autism. 2014 Oct 27;5:51. doi: 10.1186/2040-2392-5-51 (PMC4218990; doi:10.1186/2040-2392-5-51)

A) ASD

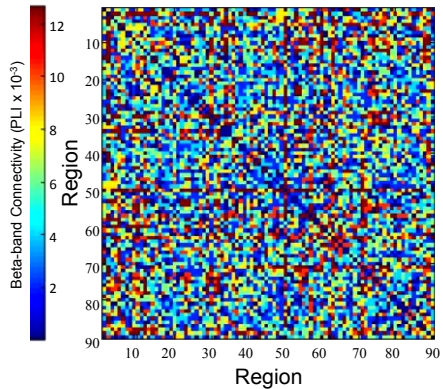

B) Controls

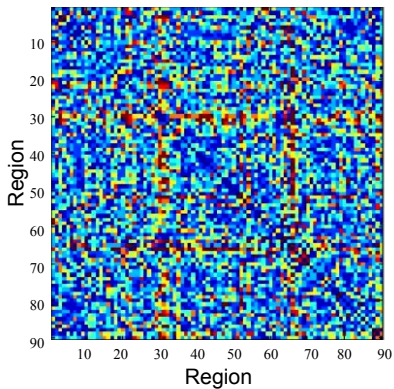

C) Difference

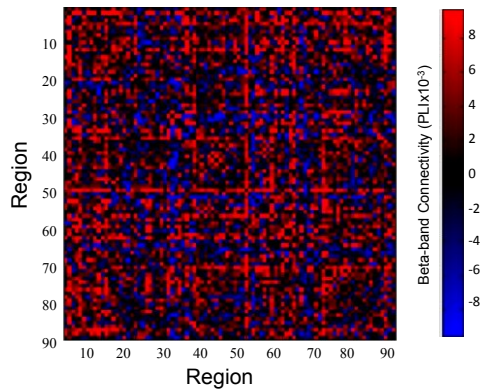

Supplement: Supplementary file 1 — Additional file 1: Figure S1: Connectivity matrices in the beta frequency band during angry face processing. Group averages of connectivity dynamics in the beta frequency band in response to angry faces during angry face processing in A) adolescents with ASD and B) controls. C) Between-group differences in connectivity dynamics highlights the disorganization in connectivity dynamics in ASD. See Additional file 2: Table S1 for regions, coordinates, and corresponding labels. (PDF 298 KB) [file 13229_2014_141_MOESM1_ESM.pdf]
