# Supplementary material for: Reduced beta connectivity during emotional face processing in adolescents with autism
Source: Mol Autism. 2014 Oct 27;5:51. doi: 10.1186/2040-2392-5-51 (PMC4218990; doi:10.1186/2040-2392-5-51)
Supplement: Supplementary file 2 — Additional file 2: Table S1: Showing regions, locations, and corresponding labels for connectivity matrices in Additional file 1: Figure S1. (DOCX 24 KB) [file 13229_2014_141_MOESM2_ESM.docx]

**Additional file 2**

**Supplemental Table 1. Regions, locations, and corresponding labels for connectivity matrices in Supplemental Figure 1. Coordinates are shown in MNI space.**

| Region | x | y | z | Label |
| --- | --- | --- | --- | --- |
| 1 | -40 | -6 | 51 | Precentral_L |
| 2 | 40 | -8 | 52 | Precentral_R |
| 3 | -19 | 35 | 42 | Frontal_Sup_L |
| 4 | 20 | 31 | 44 | Frontal_Sup_R |
| 5 | -18 | 47 | -13 | Frontal_Sup_Orb_L |
| 6 | 17 | 48 | -14 | Frontal_Sup_Orb_R |
| 7 | -34 | 33 | 35 | Frontal_Mid_L |
| 8 | 37 | 33 | 34 | Frontal_Mid_R |
| 9 | -32 | 50 | -10 | Frontal_Mid_Orb_L |
| 10 | 32 | 53 | -11 | Frontal_Mid_Orb_R |
| 11 | -49 | 13 | 19 | Frontal_Inf_Oper_L |
| 12 | 49 | 15 | 21 | Frontal_Inf_Oper_R |
| 13 | -47 | 30 | 14 | Frontal_Inf_Tri_L |
| 14 | 49 | 30 | 14 | Frontal_Inf_Tri_R |
| 15 | -37 | 31 | -12 | Frontal_Inf_Orb_L |
| 16 | 40 | 32 | -12 | Frontal_Inf_Orb_R |
| 17 | -48 | -8 | 14 | Rolandic_Oper_L |
| 18 | 52 | -6 | 15 | Rolandic_Oper_R |
| 19 | -6 | 5 | 61 | Supp_Motor_Area_L |
| 20 | 8 | 0 | 62 | Supp_Motor_Area_R |
| 21 | -9 | 15 | -12 | Olfactory_L |
| 22 | 8 | 16 | -11 | Olfactory_R |
| 23 | -6 | 49 | 31 | Frontal_Sup_Medial_L |
| 24 | 8 | 51 | 30 | Frontal_Sup_Medial_R |
| 25 | -6 | 54 | -7 | Frontal_Med_Orb_L |
| 26 | 7 | 52 | -7 | Frontal_Med_Orb_R |
| 27 | -6 | 37 | -18 | Rectus_L |
| 28 | 7 | 36 | -18 | Rectus_R |
| 29 | -36 | 7 | 3 | Insula_L |
| 30 | 38 | 6 | 2 | Insula_R |
| 31 | -5 | 35 | 14 | Cingulum_Ant_L |
| 32 | 7 | 37 | 16 | Cingulum_Ant_R |
| 33 | -6 | -15 | 42 | Cingulum_Mid_L |
| 34 | 7 | -9 | 40 | Cingulum_Mid_R |
| 35 | -6 | -43 | 25 | Cingulum_Post_L |
| 36 | 6 | -42 | 22 | Cingulum_Post_R |
| 37 | -26 | -21 | -10 | Hippocampus_L |
| 38 | 28 | -20 | -10 | Hippocampus_R |
| 39 | -22 | -16 | -21 | ParaHippocampal_L |
| 40 | 24 | -15 | -20 | ParaHippocampal_R |
| 41 | -24 | -1 | -17 | Amygdala_L |
| 42 | 26 | 1 | -18 | Amygdala_R |
| 43 | -8 | -79 | 6 | Calcarine_L |
| 44 | 15 | -73 | 9 | Calcarine_R |
| 45 | -7 | -80 | 27 | Cuneus_L |
| 46 | 13 | -79 | 28 | Cuneus_R |
| 47 | -16 | -68 | -5 | Lingual_L |
| 48 | 15 | -67 | -4 | Lingual_R |
| 49 | -18 | -84 | 28 | Occipital_Sup_L |
| 50 | 23 | -81 | 31 | Occipital_Sup_R |
| 51 | -33 | -81 | 16 | Occipital_Mid_L |
| 52 | 36 | -80 | 19 | Occipital_Mid_R |
| 53 | -37 | -78 | -8 | Occipital_Inf_L |
| 54 | 37 | -82 | -8 | Occipital_Inf_R |
| 55 | -32 | -40 | -20 | Fusiform_L |
| 56 | 33 | -39 | -20 | Fusiform_R |
| 57 | -43 | -23 | 49 | Postcentral_L |
| 58 | 40 | -25 | 53 | Postcentral_R |
| 59 | -24 | -60 | 59 | Parietal_Sup_L |
| 60 | 25 | -59 | 62 | Parietal_Sup_R |
| 61 | -44 | -46 | 47 | Parietal_Inf_L |
| 62 | 45 | -46 | 50 | Parietal_Inf_R |
| 63 | -57 | -34 | 30 | SupraMarginal_L |
| 64 | 57 | -32 | 34 | SupraMarginal_R |
| 65 | -45 | -61 | 36 | Angular_L |
| 66 | 45 | -60 | 39 | Angular_R |
| 67 | -8 | -56 | 48 | Precuneus_L |
| 68 | 9 | -56 | 44 | Precuneus_R |
| 69 | -9 | -25 | 70 | Paracentral_Lobule_L |
| 70 | 6 | -32 | 68 | Paracentral_Lobule_R |
| 71 | -12 | 11 | 9 | Caudate_L |
| 72 | 14 | 12 | 9 | Caudate_R |
| 73 | -25 | 4 | 2 | Putamen_L |
| 74 | 27 | 5 | 2 | Putamen_R |
| 75 | -19 | 0 | 0 | Pallidum_L |
| 76 | 20 | 0 | 0 | Pallidum_R |
| 77 | -12 | -18 | 8 | Thalamus_L |
| 78 | 12 | -18 | 8 | Thalamus_R |
| 79 | -43 | -19 | 10 | Heschl_L |
| 80 | 45 | -17 | 10 | Heschl_R |
| 81 | -54 | -21 | 7 | Temporal_Sup_L |
| 82 | 57 | -22 | 7 | Temporal_Sup_R |
| 83 | -41 | 15 | -20 | Temporal_Pole_Sup_L |
| 84 | 47 | 15 | -17 | Temporal_Pole_Sup_R |
| 85 | -57 | -34 | -2 | Temporal_Mid_L |
| 86 | 56 | -37 | -1 | Temporal_Mid_R |
| 87 | -37 | 15 | -34 | Temporal_Pole_Mid_L |
| 88 | 43 | 15 | -32 | Temporal_Pole_Mid_R |
| 89 | -51 | -28 | -23 | Temporal_Inf_L |
| 90 | 53 | -31 | -22 | Temporal_Inf_R |
